# Antifungal activity and mycotoxin-inhibiting potential of amphisin and rhamnolipids from *Pseudomonas* strains

**DOI:** 10.1038/s41598-025-31914-1

**Published:** 2025-12-13

**Authors:** Dominika Ciurko, Aleksandra Grzywacz, Kinga Hyla, Anna Kancelista, Tomasz Janek

**Affiliations:** 1 Department of Physics and Biophysics, Wrocław University of Environmental and Life Sciences, 50-375 Wrocław, Poland; 2 Department of Biotechnology and Food Microbiology, Wrocław University of Environmental and Life Sciences, 51-630 Wrocław, Poland

**Keywords:** *Pseudomonas* biosurfactants, Amphisin, Rhamnolipids, Antifungal activity, Plant pathogens, Post-harvest protection, Mycotoxin contamination, Antimicrobials, Fungi

## Abstract

Biosurfactants, due to their amphiphilic properties, are widely used across a broad spectrum of industries. In this paper, we describe experiments conducted to determine the antagonistic activity of amphisin and rhamnolipids against fungal plant pathogens. The bacterial strains used for biosurfactants production were *Pseudomonas fluorescens* DSS73 and *Pseudomonas aeruginosa* #112. Both amphisin and rhamnolipids were produced using waste raw materials from the food industry. The biosurfactants exhibited significant antifungal activity and the ability to reduce sterigmatocystin production. The most significant inhibition was observed during the initial days of the experiments, followed by a gradual decrease in biosurfactants activity. However, a long-term effect was obtained against *Aspergillus amoenus* DSM 1943, *Penicillium nordicum* DSM 12639, and *Monilinia fructigena* IOR 2138. Additionally, sterigmatocystin production in cultures of *A. amoenus* DSM 1943 and *Aspergillus quadrilineatus* DSM 820 was reduced by 89–99%, with the most significant results observed for rhamnolipids. Therefore, as demonstrated in this research, *Pseudomonas*-derived biosurfactants show great potential in plant cultivation by protecting against fungal invasion. Amphisin and rhamnolipids can be successfully applied as eco-friendly solutions to limit sterigmatocystin accumulation during crop storage.

## Introduction

Pathogenic fungi are major contributors to food loss globally, causing infection of seeds and growing crops as well as spoilage of post-harvest and in processed foods, during manufacturing, transport and storage. It is estimated that phytopathogenic fungi are responsible for up to 20% loss of global crop yield, which is enough to feed up to 600 million people annually. In addition, some spoilage fungi pose a potential threat to life and health through the production of mycotoxins or by infecting hosts with reduced immunity^1^. According to the ranking of a journal Molecular Plant Pathology *Botrytis*, some of *Fusarium* and *Colletotrichum* spp. has been nominated to ‘Top 10’ fungal plants pathogens based on scientific/economic importance^2^.

*Botrytis* spp. is a group of fungi incorporating several plant pathogens that are hosted by approximately 1400 plant species. The most destructive, *Botrytis. cinerea*, is the agent of grey mould. It is classified as necrotroph able to infect stems, leaves, and fruits. Due to the wide range of its host, it is difficult to estimate the economic losses caused by the fungus. However, it is assumed to be millions of dollars annually, depending on the agricultural sector^3^.

Over the years, the *Fusarium* genus has become economically significant due to the capital loss it generates as a plant pathogen. The genus *Fusarium* is responsible for diseases such as crown rot, head blight, and scab on cereal grains. In addition, it triggers vascular wilts on a wide range of horticultural crops (tomato, banana, and others), root rots, and cankers among others^4^. *Fusarium graminearum* and *Fusarium culmorum* are the two predominant species infecting wheat. In warmer and more humid regions of the world, such as the United States of America (USA), *F. graminearum* is recognized as the main infection factor, while *F. culmorum* dominates in cooler areas, such as Northern and Central Europe, as well as Canada. In addition, *F. culmorum* is capable to product wide spectrum of mycotoxins, such as the trichothecenes (deoxynivalenol (DON), nivalenol (NIV), 3-acetyldeoxynivalenol and acetyl T-2 toxin), zearalenone (ZON) and fusarins therefore lot of attention is pay into developing its biocontrol methods^5^.

*Colletotrichum* is a large genus of the *Ascomycetes* including over 190 species infecting a wide variety of crops in the tropical, subtropical, and temperate regions causing plant disease known as ‘‘anthracnose’’. Infection symptoms are described as a sunken necrotic plant tissue in which numerous orange conidia are produced^6^. Moreover, *Colletotrichum* contributes to other plant diseases such as straw berry and banana crown rot, red rot in coffee berries and sugar cane, blotch in cowpea as well as black dot of potato. The most important species, leading to huge economic losses, are *Colletotrichum graminicola* affecting maize, rice and wheat production, *Colletotrichum higginsianum* being the pathogen of kales, cabbages and broccoli, *Colletotrichum trifolii* and *Colletotrichum lindemuthianum* hosted by alfalfa and legumes respectively as well as *Colletotrichum coccodes* infected potato^7,8^.

Not without the economic significance are fungi classified to the genus *Aspergillus*, which is widely distributed in nature thanks to the size and easy dispersion of conidia. It is considered to be responsible for the majority of agricultural pollution and as main agents being behind mycotoxin contamination of crops. Under certain circumstances *Aspergillus*, may be hazardous to human, causing opportunistic infection^9^.

In economic terms genus *Monilinia* are one of the most important limiting factors for fruit production all over the world. Symptoms such as blossom, twig and branch blight, as well as fruit rot, observed mostly on the *Rosaceae* family are the main indicator of *Monilinia* infection. Three species are considered to be economically significant: *M. fructigena*, found mainly on pome fruits, *Monilinia laxa* and *Monilinia fructicola* causing disease of stone fruits^10^.

Another genus having a large economic impact on human life is *Penicillium. Penicillium* spp. are one of the most common fungi, having worldwide distribution, being detected in soil, on the vegetation and in the air and water. A large number of studies report the positive impact of *Penicillium* spp. on crop roots, enhancing plant growth^11^. Some *Penicillium* spp. have antagonistic activity against plant pathogens^12^. In contrary, others contribute to the diseases of plants and animals. *Penicillium expansum*,* Penicillium verrucosum*,* Penicillium italicum*,* Penicillium digitatum and Penicillium citrinum*, are only few examples of important disease triggers being behind pre- and postharvest agricultural losses. *Penicillium* spp. are responsible for pre- and postharvest pathogenesis such as fruit rot in citrus, kernel rot in cashews, and blue mold in pear, peach, and apple^13^.

Commercial agriculture is mainly based on the application of chemical fungicides to protect crop plants against fungal pathogens. However, fungicides are commonly overuse or misuse, which in turn leads to their toxicity in relation to beneficial living systems, human and animals. Moreover, it contributes to the continuous development of resistance, making fungal pathogens increasingly difficult to treat. Therefore, there is an urgent need to develop healthy, non-toxic, and eco-friendly alternative approaches^14^.

Biosurfactants are microbial secondary metabolites that contain both hydrophobic and hydrophilic groups within a single molecule, making them amphiphilic^15^. Due to properties, biosurfactants have substantial potential in environmental applications such as bioremediation, wastewater and sludge treatment as well as in pharmaceutical and petroleum industries^16^. However, it is worth emphasizing that biosurfactants are increasingly gaining attention due to the growing number of scientific reports indicating their effectiveness in plant biocontrol because of their antimicrobial activity^17^. When compared to their chemical counterparts, biosurfactants offers numerous benefits including biodegradability, bioavailability, biocompatibility, high selectivity, environmental suitability, and greater efficiency under higher temperatures and salinity stress, which makes them more attractive in terms of agricultural applications. They also have the advantages of being biologically produced in a sustainable way, using renewable resources^16,18^.

**Fig. 1:**
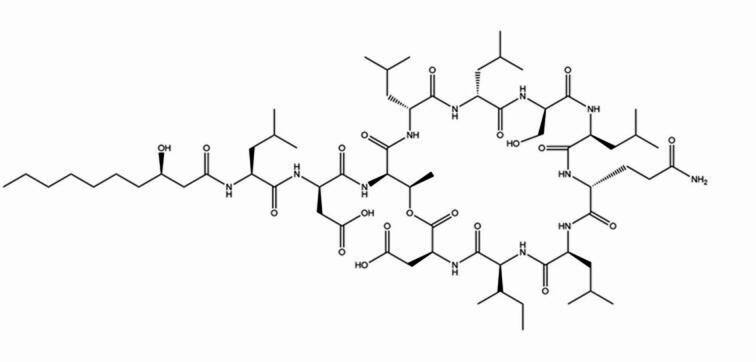
Structure of amphisin showing a β-hydroxydecanoyl fatty acid attached to an undecapeptide sequence (D-Leu-D-Asp-D-aThr-D-Leu-D-Leu-D-Ser-L-Leu-D-Gln-L-Leu-L-Ile-L-Asp).

Amphisin, being classified to the group of lipopeptide biosurfactants, deserves special attention, thanks to its significant antibacterial and antifungal feature. It is a cyclic lipoundecapeptide, linked to at a *3R*-hydroxydecanoic acid (3-OH C10:0) moiety at the N-terminus (Fig. 1). The structure of amphisin, produced by *P. fluorescens* DSS73, is mainly helical, with cyclic peptide wrapping around a hydrogen-bonded water molecule^19^.

Another popular group of biosurfactants, being in the center of scientific interest due to the inhibitory activity against microbiological pathogen are rhamnolipids. Rhamnolipid biosurfactants are primarily produced by *P. aeruginosa* and consist of one or two rhamnose sugars linked to β-hydroxy fatty acids^20^. Rhamnolipids have diverse spectrum of applications in agriculture, bioremediation, food processing and pharmaceuticals due to their excellent surface activity and biological properties^21^. Moreover, there are numerous reports indicating their biocontrol activity^21^.

Due to the growing interest in eco-friendly compounds with antimicrobial and antifungal activity, the aim of this work was to analyze, whether natural surfactants, may inhibit the growth of the fungal plant pathogens. In this work, we investigated the effects of amphisin and rhamnolipid, produced respectively in the culture of *P. fluorescens* DSS73 and *P. aeruginosa* #112, on the growth inhibition of important pre- or post-harvest pathogens *A. quadrilineatus* DSM 820, *A. amoenus* DSM 1943, *B. cinerea* IOR 663, *C. coccodes* DSM 62126, *F. culmorum* DSM 1094, *M. fructigena* IOR 2138, *P. expansum* CBS 230.38 and *P. nordicum* DSM 12639. In addition, due to the significant harmfulness to humans and animals, as well as the high economic cost of crops pollution, we determined, if *Pseudomonas* amphisin and rhamnolipids can affect the production of sterigmatocystin by *A. amoenus* DSM 1943 and *A. quadrilineatus* DSM 820.

## Results and discussion

### Biosurfactants productivity and rhamnolipids profile

In this work, the concentration of amphisin in the culture of *P. fluorescens* DSS73 reach 16.22 ± 0.25 g/L. Obtained result correspond well with our previous work (16.51 ± 0.49 g/L), where the optimal conditions for lipopeptide production was determined^22^. The amount of rhamnolipids recovered at the end of the fermentation (120 h) was 1424 ± 26 mg/L. Eight rhamnolipid homologs were identified, among which four di-rhamnolipids and four mono-rhamnolipids were detected. The ratio of di- to mono-rhamnolipids was 38.41:61.59 with mono-rhamnolipid Rha-C_10_-C_10_ being the most abundant, making up 45.29% of the total content (Table 1). Moreover, Rha-Rha-C_10_-C_10_ had a significant share in the total pool of produced rhamnolipids, constituting of 26.18%.

Patel and Desai^23^ described the production of rhamnolipids by a *P. aeruginosa* GS3 utilizing a medium composed of molasses (7% (v/v)) and corn steep liquor (0.5% (v/v)). However, despite similar medium composition, the quantity of rhamnolipids produced was significantly lower (240 mg/L) compared to our research. In another studies Silva et al.^24^ obtained significant rhamnolipids production, up to 4.15 g/L culturing *Pseudomonas* sp. P3 on the medium supplemented with molasses and corn steep liquor in various proportions. Another significant results were obtained by Gudiña et al.^25^ when *P. aeruginosa* #112, cultured on the medium composed of corn steep liquor (10%, v/v), sugarcane molasses (10%, w/v) and olive mill wastewater (25%, w/v), produced up to 4.526 g/L and 5.124 g/L rhamnolipids, respectively in the flask and reactor. In correlation to our studies, the most abundant, among seven observed homologues, was mono-rhamnolipid Rha-C_10_–C_10_, followed by the di-rhamnolipid Rha-Rha-C_10_–C_10_. In agreement with our findings, mono-rhamnolipids were markedly predominant over di-rhamnolipids, exhibiting mono-to-di ratios of 1.67 and 2.37 in flask and reactor cultures, respectively.

In summary, the production efficiency and the proportion of rhamnolipid homologues are influenced by many factors, the most important of which are the strain used, the composition of the medium, and the relative proportions of the medium components.

**Table 1:** Rhamnolipid homologues distribution in the culture of *P. aeruginosa* #112.

Rhamnolipid homologues		Relative abundance %
Mono-rhamnolipids		
Rha-C_10_-C_8_		5.34
Rha-C_10_-C_10_		45.29
Rha-C_10_-C_12:1_		4.71
Rha-C_10_-C_12_		6.25
Di-rhamnolipids		
Rha-Rha-C_8_-C_10_		1.72
Rha-Rha-C_10_-C_10_		26.18
Rha-Rha-C_10_-C_12:1_		3.85
Rha-Rha-C_10_-C_12_		6.66

### Antifungal activity of biosurfactants against filamentous fungi

In a first stage of the research, the antifungal activity of amphisin and rhamnolipids were examined in relation to *Aspergillaceae* family. Significant activity of amphisin, applied in two concentration 0.3 g/L and 0.5 g/L were determined against to *A. quadrilineatus* DSM 820, *A. amoenus* DSM 1943, *P. nordicum* DSM 12639 and *P. expansum* CBS 230.38 (Table 2).

**Table 2:** The average mycelium inhibition rates (%) of *A. quadrilineatus* DSM 820, *A. amoenus* DSM 1943, *P. nordicum* DSM 12639 and *P. expansum* CBS 230.38.

Amphisin [g/L]								
	*A. quadrilineatus* DSM 820		*A. amoenus* DSM 1943		*P. nordicum* DSM 12639		*P. expansum* CBS 230.38	
	0.3	0.5	0.3	0.5	0.3	0.5	0.3	0.5
48 h	**27.34 ± 8.14**	**35.14 ± 5.23**	–	–	–	–	13.99 ± 5.46	20.56 ± 4.49
72 h	17.71 ± 3.66	25.96 ± 2.8	–	–	**100**	**100**	**18.29 ± 4.38**	23.30 ± 2.31
120 h	22.3 ± 1.83	24.17 ± 2.44	**56.08 ± 3.14**	**39.77 ± 17.11**	36.89 ± 1.04	45.53 ± 3.45	14.86 ± 2.71	**23.79 ± 2.27**
168 h	12.94 ± 0.89	11.17 ± 1.01	46.91 ± 3.44	34.96 ± 9.65	36.06 ± 9.57	46.11 ± 4.86	13.91 ± 1.67	19.22 ± 4.27
216 h	23.79 ± 1.17	22.91 ± 0.64	45.28 ± 9.25	28.86 ± 10.37	30.01 ± 4.79	34.88 ± 3.75	11.53 ± 2.52	15.49 ± 0.99
264 h	nh	nh	40.56 ± 3.13	28.27 ± 8.38	31.65 ± 4.28	35.39 ± 4.30	nh	nh
312 h	nh	nh	29.14 ± 6.83	23.90 ± 9.85	30.65 ± 3.59	36.76 ± 2.87	nh	nh

Growth inhibition was observed on media supplemented with amphisin and compared to the control.–, lack of the fungal grow both in the control and tested sample; 100, lack of the fungal growth compared to the control; nh, growth inhibition has no longer been observed. Significant values are in [bold].

Generally, higher inhibition of the fungal growth was observed, when the media was supplemented with 0.5 g/L of amphisin. The exception to the rule was the analysis performed with *A. amoenus* DSM 1943. In this case, amphisin, applied in the concentration of 0.3 g/L were much more effective compared to the higher dose. In addition, the most significant inhibition effect was observed in the initial days of incubation, then antifungal activity gradually decreased. In the culture of *P. nordicum* DSM 12639, the degree of mycelium inhibition rates declined from 100% (at 72 h of cultivation) to approximately 30–35% (312 h) depend on the concentration applied. The reason for the drop in biosurfactant activity has not been investigated until now. We hypothesize it can be due to adaptation of fungi to the environmental changes by producing biosurfactant degrading enzymes^26^ or altering their cell membranes to reduce biosurfactant toxicity^27^. However, a precise explanation of the mechanism by which filamentous fungi develop resistance to biosurfactants over time requires additional research and must be clarified in the future. Amphisin showed significant activity against *A. amoenus* DSM 1943. After 120 h of incubation, the fungal growth was inhibited by 56.08% and 39.77% respectively for the concentration of 0.3 g/L and 0.5 g/L. Obtained results indicate the possibility of using amphisin in agricultural or storage practice as an agent limiting the development of *Aspergillaceae* pathogens. However, there is a need to explain and overcome the problem of time increasing fungal resistance.

Antagonistic activity of *Pseudomonas* biosurfactants has been examined before by Schlusselhuber et al.^28^. Close relative of amphisin lipopeptide, milkisin, showed weak antifungal activity against *P. expansum* CMPG 136 and lack of activity in relation to *Aspergillus niger* CMPG 814. These results are in contrast to our studies, where significant activity of amphisin against both * Penicillium and Aspergillus* species was confirmed. In our previous study, we confirmed antagonistic activity of amphisin in relation to *A. carbonarius* MUM 05.18 and we excluded the possibility of using amphisin as a biocontrol agent against *A. flavus* MUM 17.14 and *A. niger* MUM 92.13^22^. In this research we extend the spectrum of amphisin activity by *A. quadrilineatus* DSM 820 and *A. amoenus* DSM 1943. The effect of rhamnolipids on the growth of fungi, representing *Aspergillaceae* family was as good or better as in case of amphisin **(**Table 3**)**.

**Table 3:** The average mycelium inhibition rates (%) of *A. quadrilineatus* DSM 820, *A. amoenus* DSM 1943, *P. nordicum* DSM 12639 and *P. expansum* CBS 230.38.

Rhamnolipids [g/L]								
	*A. quadrilineatus* DSM 820		*A. amoenus* DSM 1943		*P. nordicum* DSM 12639		*P. expansum* CBS 230.38	
	1.5	3	1.5	3	1.5	3	1.5	3
48 h	–	–	–	–	–	–	–	–
72 h	**77.67 ± 1.24**	**86.86 ± 3.73**	–	–	**54.89 ± 5.87**	**57.23 ± 7.88**	**50.66 ± 1.39**	49.93 ± 3.14
120 h	72.90 ± 1.92	80.64 ± 0.43	–	–	38.92 ± 3.16	31.20 ± 3.58	34.44 ± 1.56	45.42 ± 2.41
168 h	66.92 ± 2.04	74.15 ± 1.09	41.80 ± 3.76	51.50 ± 3.64	18.71 ± 1.79	20.73 ± 3.93	46.98 ± 4.24	**54.38 ± 2.40**
216 h	nh	nh	**44.22 ± 4.65**	**54.80 ± 2.54**	36.32 ± 3.12	26.89 ± 4.31	nh	nh
264 h	nh	nh	28.31 ± 0.18	35.55 ± 4.30	31.52 ± 4.58	19.11 ± 2.16	nh	nh
312 h	nh	nh	30.83 ± 1.79	27.52 ± 1.87	33.05 ± 5.59	22.21 ± 3.10	nh	nh

Growth inhibition was observed on media supplemented with rhamnolipids and compared to control.–, lack of the fungal grow both in the control and tested sample; nh – growth inhibition has no longer been observed. Significant values are in [bold].

Likewise, the highest inhibition of the fungal growth was observed in the initial days of the experiment. Supplementation with rhamnolipids was the most effective in relation to *A. quadrilineatus* DSM 820, where the mycelium growth was inhibited in 77.67% and 86.86% respectively for the concentration of 1.5 g/L and 3 g/L. In case of *P. expansum* CBS 230.38 the addition of rhamnolipids to the culture medium has a stronger antifungal effect than that, observed for amphisin.

Studies, confirming inhibitory activity of rhamnolipids against *Aspergillus* species were presented before by Rodrigues et al.^29^. Partially purified rhamnolipids mixture at a concentration of 3 g/L inhibited the growth of *A. niger* MUM 92.13 and *A. carbonarius* MUM 05.18 in 28.0% and 22.6% respectively. Our studies confirmed results obtained before, extending the spectrum of rhamnolipids activity.

In the study by Rodrigues et al.^30^, the effect of rhamnolipids on the growth of *A*. *flavus* MUM 17.14 was examined over a concentration range of 45–1500 mg/L. Rhamnolipids only partially inhibited fungal growth, and, in contrast to our findings, no clear relationship between biosurfactant concentration and growth inhibition was observed. These results indicate the absence of a uniform pattern of action within the *Aspergillaceae* family, suggesting that the antagonistic activity of rhamnolipids is strain-specific.

**Fig. 2:**
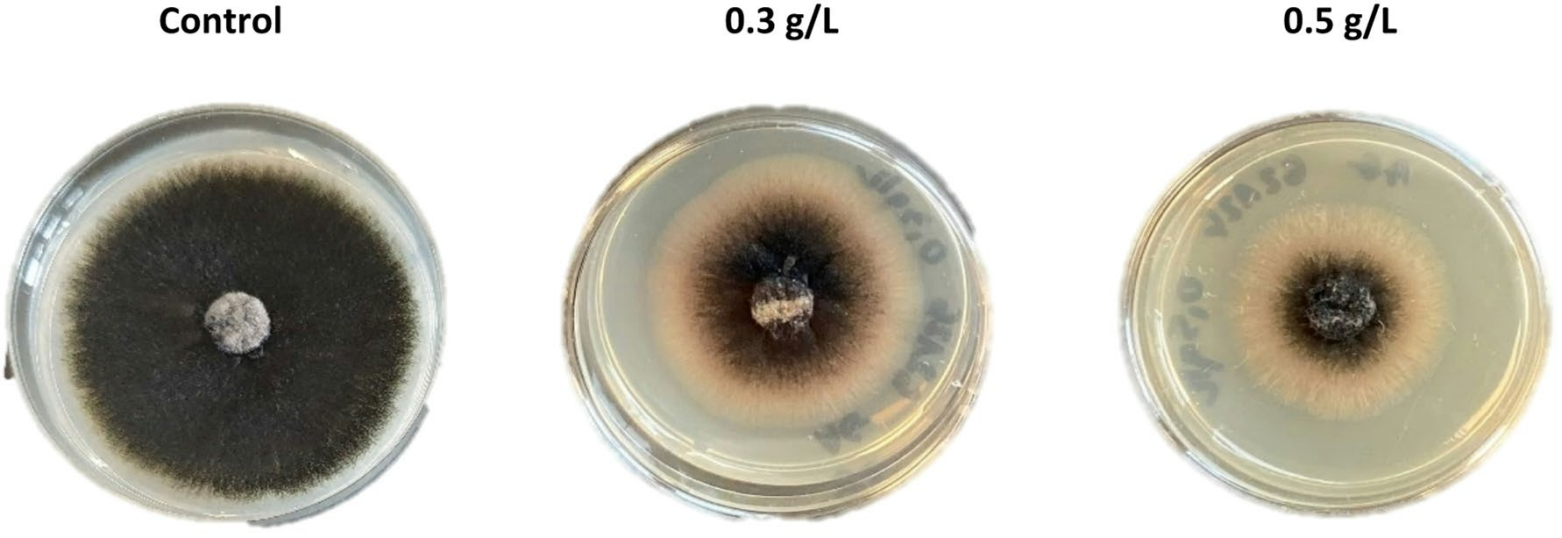
Inhibition of growth and sporulation of *C. coccodes* DSM 62126 observed on the media supplemented with 0.3 g/L and 0.5 g/L amphisin.

In the next stage of the research the activity of amphisin and rhamnolipids were determined against two species of *Sclerotiniaceae* family *B. cinerea* and *M. fructigena* as well as *F. culmorum* being behind plant fusariosis and *C. coccodes* causing tomato and potato anthracnose. Amphisin exhibited very strong antifungal activity relative to *M. fructigena* IOR 2138 (Table 4). In addition, we noticed relevant inhibition of *B. cinerea* IOR 663 growth, respectively 55.37% and 58.66% for the concentration of 0.3 g/L and 0.5 g/L. As it was observed among *Aspergillaceae* family, amphisin was the most effective in the initial days of cultures, then its activity decrease. For example, in the culture of *B. cinerea* IOR 663 and *F. culmorum* DSM 1094, after 72 h of incubation, the inhibitory effect has been abolished. In the culture of *C. coccodes* DSM 62126, inhibition of fungal growth and sporulation was observed (Fig. 2).

**Table 4:** The average mycelium inhibition rates (%) of *B. cinerea* IOR 663, *M. fructigena* IOR 2138, *F. culmorum* DSM 1094 and *C. coccodes* DSM 62126.

Amphisin [g/L]								
	*B. cinerea* IOR 663		*M. fructigena* IOR 2138		*F. culmorum* DSM 1094		*C. coccodes* DSM 62126	
	0.3	0.5	0.3	0.5	0.3	0.5	0.3	0.5
48 h	**55.37 ± 2.71**	**58.66 ± 1.59**	–	–	23.4 ± 3.56	15.93 ± 8.09	**28.44 ± 3.01**	**24.99 ± 1.75**
72 h	48.69 ± 2.57	50.22 ± 1.68	**100**	**100**	**28.91 ± 1.09**	**38.87 ± 1.5**	26.17 ± 1.24	24.31 ± 1.94
120 h	nh	nh	41.98 ± 3.21	62.29 ± 2.49	nh	nh	15.86 ± 1.01	17.48 ± 1.37
168 h	nh	nh	39.23 ± 6.43	46.82 ± 1.11	nh	nh	nh	nh
216 h	nh	nh	43.25 ± 4.97	47.54 ± 2.62	nh	nh	18.01 ± 1.45	19.80 ± 0.89
264 h	nh	nh	41.68 ± 3.83	43.37 ± 2.05	nh	nh	nh	nh
312 h	nh	nh	40.32 ± 5.32	40.49 ± 2.57	nh	nh	nh	nh

Growth inhibition was observed on media supplemented with amphisin and compared to control.–, lack of the fungal grow both in the control and tested sample; 100, lack of the fungal growth compared to the control; nh, growth inhibition has no longer been observed. Significant values are in [bold].

Antagonistic effect of *Pseudomonas* lipopeptides against *B. cinerea* strain DAOM 189,076 were examined in the work of Kurniawan et al.^31^. Purified extracts of *Pseudomonas arsenicoxydans* F9-7, *Pseudomonas koreensis* F9-9, *Pseudomonas brenneri* F9-10, and *Pseudomonas moraviensis* strain F9-12 post-culture media showed marked inhibition of spore germination (averaging 92% inhibition). When these same extracts were assessed for inhibition of mycelia growth antifungal activity was observed only in case of *P. koreensis* F9-9 and *P. arsenicoxydans* F9-7. Qualitative analysis resulted in detection of arthrofactin, lipopeptide belonging to the amphisin family, and its two homologs as a compound responsible for antifungal activity. Presented research confirmed antagonistic activity of amphisin family in relation to *B. cinerea* species, which was detected in our studies also indicating inhibition of spore germination as a possible mechanism of lipopeptides action.

However, many other possible mechanisms of biosurfactants activity have been demonstrated to this day. Lipopeptides may induced the increased generation of reactive oxygen species (ROS), causing serious damage to the cell wall and cytoplasm^16^. They may alter fungal cell wall integrity and permeability^32^ as well as inhibit the fungal growth by interfering with glycolysis/gluconeogenesis and the tricarboxylic acid cycle by reducing succinate (SDH) and malate dehydrogenase (MDH) as well ATPase activity. Finally, lipopeptides may influence the expression of a fungal proteins impairing among the other amino acid biosynthesis and degradation, metabolism of 2-oxocarboxylic acid, carbon monoxide, glyoxylate and dicarboxylate as well as biosynthesis of pantothenate and Coenzyme A^33^. Therefore, we are in favor of the hypothesis that lipopeptides have board cellular effect and affect wide array of cellular functions and therefore the sum of these processes underlies the amphisin antifungal activity. Significant antagonistic activity in relation to *M. fructigena* IOR 2138 and *C. coccodes* DSM 62126 where observed when the media were supplemented with rhamnolipids **(**Table 5**)**. In the culture of *B. cinerea* IOR 663 and *F. culmorum* DSM 1094, antagonistic activity was observed up to 72 h of incubation Then, antifungal activity of biosurfactants declined. Both tests conducted with the addition of amphisin as well as rhamnolipids showed the prospective action of biosurfactants, due to the long-term effect, against *M. fructigena* IOR 2138. We believe that long term inhibition of the growth of remaining tested strain is possible, but may require the administration of another dose of biosurfactants. In our opinion stable, continuous plant protective effect is of great importance when the integration of biosurfactants into existing agricultural practices is considered.

The antifungal activity of rhamnolipids against *Colletotrichum orbiculare* has been determined before in the work of Andrieu et al.^34^. At the concentration of 50 µg/mL rhamnolipid B did not affect hyphal growth, however effectively inhibited spore germination, what can be one of the putative mechanism of antagonistic activity of rhamnolipids. In contrast to our research, rhamnolipid B has been not active in relation to *B. cinerea.* The same effect was observed *against Fusarium oxysporum f. sp. cucumerinum*, while in our study significant inhibition of *F. culmorum* DSM 1094 were determined^35,36^.

**Table 5:** The average mycelium inhibition rates (%) of *B. cinerea* IOR 663, *M. fructigena* IOR 2138, *F. culmorum* DSM 1094 and *C. coccodes* DSM 62126.

Rhamnolipids [g/L]								
	***B. cinerea*** **IOR 663**		***M. fructigena*** **IOR 2138**		***F. culmorum*** **DSM 1094**		***C. coccodes*** **DSM 62126**	
	1.5	3	1.5	3	1.5	3	1.5	3
48 h	44.25 ± 3.55	**64.41 ± 5.46**	–	–	**62.07 ± 1.74**	**70.38 ± 3.04**	–	–
72 h	**56.24 ± 4.87**	60.77 ± 7.17	**62.86 ± 0.16**	**68.99 ± 1.03**	57.72 ± 0.63	66.73 ± 1.30	63.31 ± 3.25	**75.31 ± 4.70**
120 h	nh	nh	62.69 ± 0.00	62.94 ± 4.58	nh	Nh	**63.97 ± 3.58**	70.88 ± 2.79
168 h	nh	nh	49.46 ± 6.48	51.12 ± 4.83	nh	Nh	49.42 ± 4.78	59.69 ± 2.50
216 h	nh	nh	46.88 ± 6.46	46.38 ± 2.00	nh	Nh	nh	nh
264 h	nh	nh	46.09 ± 2.86	41.31 ± 0.56	nh	Nh	nh	nh
312 h	nh	nh	42.20 ± 4.03	39.42 ± 0.76	nh	Nh	nh	nh

Growth inhibition was observed on media supplemented with rhamnolipids and compared to control.–, lack of the fungal grow both in the control and tested sample; 100, lack of the fungal growth compared to the control; nh, growth inhibition has no longer been observed. Significant values are in [bold].

In another studies rhamnolipids produced by *P. aeruginosa* SS14 were applied as an efficient agent against *Fusarium verticillioides* FS7^37^. At the concentration of 200 mg/L the growth of the fungus was totally inhibited. Treated mycelia, besides being reduced in thickness, exhibited irregular shape with uneven surface and breakage. Destruction of mycelium may lie as well at the bottom of *F. culmorum* DSM 1094 inhibition observed in our studies.

Summarizing, as in the case of lipopeptides several processes, underlying the inhibition of fungal growth by rhamnolipids, have been proposed to this day, among which inhibition of spore germination^34^ and changes in hyphal morphology such as breakage, irregular shape and reduced thickness have been mentioned^37^. We supposed, that no single mechanism is responsible for inhibiting the growth of the fungus, but it always results from the synergistic action of simultaneously occurring processes. In fact, most of research on the antifungal activity of biosurfactant underline association of inhibitory activity with the damage of the cell-wall structure of the hyphae and spores, causing multiple metabolic disorders resulting from the disruption of the molecular mechanisms of fungi.

Studies of a great importance, explaining key mechanisms of rhamnolipids action, have been performed by Robineau et al.^38^. Structure–function analysis revealed that the chain length of the lipid moiety is critical for rhamnolipid activity. In the presence of the synthetic C_10_ rhamnose ester, germ tube formation and development of *B. cinerea* Pers. T4 were completely inhibited, whereas the C_12_ ester exerted a less pronounced effect. Among the tested compounds, C_10_ and C_12_ rhamnose esters induced the greatest reduction in mycelial growth, while C_12_ was the most effective in reducing infection symptoms, likely through priming the expression of the well-known defense marker gene *PRP69*. In our study, C_10_ and C_12_ rhamnolipids were the dominant homologs in the tested mixture, which explains the high inhibitory activity observed against *B. cinerea* IOR 663.

We hypothesize that the application of a mixture of rhamnolipids, as typically produced in microbial cultures, is more advantageous than the use of individual compounds. Such a mixture provides rhamnolipid esters with distinct modes of action. By applying the mixture, it is possible to simultaneously deliver compounds that inhibit hyphal growth, limit germ tube development, and strongly induce the plant immune response. In this way, a comprehensive protective barrier against the pathogen can be established.

### Effect of biosurfactants on sterigmatocystin production by *A. amoenus* DSM 1943 and *A. quadrilineatus* DSM 820

The effects of amphisin and rhamnolipids on sterigmatocystin production were studied for both concentrations (0.3 and 0.5 g/L for amphisin and 1.5 and 3.0 g/L for rhamnolipids) of biosurfactants applied. Almost complete inhibition (89.07–99.39%) of sterigmatocystin synthesis in each instance were determined **(**Table 6**).** A slightly greater reduction in mycotoxin production was noted in cultures treated with rhamnolipids. The greatest effect was determined at a concentration of 3.0 g/L, where the reduction was above 99%.

The mechanism underlying the inhibition of aflatoxin biosynthesis in the presence of rhamnolipids was proposed by Rodrigues et al.^30^. Rhamnolipids were shown to down-regulate the expression of genes *aflC*, *aflE*, *aflP*, and *aflQ*, which are involved in the aflatoxin biosynthetic pathway, leading to complete suppression of aflatoxin production. Since sterigmatocystin shares this biosynthetic pathway, we propose that its synthesis in *A. amoenus* DSM 1943 and *A. quadrilineatus* DSM 820 cultures is likely inhibited via a similar mechanism, namely through suppression of gene expression.

Numerous studies confirm the effectiveness of rhamnolipids as an agent inhibiting mycotoxin production. Our results correspond with the observations of other researchers, although some differences are apparent depending on the biosurfactants concentration and the fungal strain used.

Rhamnolipids produced by *P. aeruginosa* #112 were previously applied as an agent limiting mycotoxin production by *A. flavus* MUM 17.14^30^. At concentrations between 45 and 1500 mg/L, production of mycotoxin was inhibited by 93.9–99.5%. A more significant reduction was observed for rhamnolipids mixture, applied at concentrations up to 190 mg/L when compared with the purified mono- and di- rhamnolipids. In addition, when collating purified mono- and di- rhamnolipids, the latter were more effective above the concentration of 190 mg/L.

**Table 6:** Sterigmatocystin concentration and percentage of mycotoxin reduction in *A. amoenus* DSM 1943 and *A. quadrilineatus* DSM 820 cultures treated with biosurfactants.

Biosurfactant (BS)	Sterigmatocystin concentration (ng/mL)	Sterigmatocystin reduction (%)
*A. amoenus* DSM 1943		
Control (without BS)	3770 ± 98	–
Rhamnolipids 1.5 g/L	114 ± 18	96.97 ± 1.6
Rhamnolipids 3 g/L	23 ± 7	99.39 ± 0.3
Amphisin 0.3 g/L	412 ± 17	89.07 ± 0.6
Amphisin 0.5 g/L	213 ± 12	94.35 ± 0.6
*A. quadrilineatus* DSM 820		
Control (without BS)	1841 ± 24	–
Rhamnolipids 1.5 g/L	64 ± 4	96.52 ± 0.6
Rhamnolipids 3 g/L	12 ± 7	99.35 ± 0.3
Amphisin 0.3 g/L	156 ± 10	91.53 ± 0.3
Amphisin 0.5 g/L	76 ± 6	95.87 ± 0.3

To the best of our knowledge our research is the only one, describing the effect of *Pseudomonas* lipopeptides on mycotoxin production. In contrary, there are lot of studies concerning the inhibitory effect of *Bacillus* lipopeptide biosurfactant on mycotoxin production. In the studies performed by Veras et al.^39^ the mixture of lipopeptides, iturin A and three main isoforms of surfactin (C_13_, C_14_ and C_15_), inhibit both aflatoxin B1 (AFB1) and ochratoxin A (OTA) production. Lipopeptides produced by *Bacillus* sp. P1 and P1.1 reduced the synthesis of AFB1 in the culture of *A. flavus* A12 by 99.8%. In addition, the percentages of OTA of *A. carbonarius* ITAL293 reduction by bacteria strains were 97.5 and 97.3%, respectively. These results are quite consistent with our observations.

It is also worth mentioning the another research of Galitskaya et al.^40^ focused on the effect of *Bacillus mojavensis* PS17 lipopeptides on the production of T2 and HT2 mycotoxin. In this studies, two families of lipopeptides—fengycins and surfactins, showed efficient inhibition of toxin production by *F. oxysporum* f. sp. *lycopersici.* The obtained inhibition rate was above 80% for lipopeptide concentrations ≥ 0.5 g/L.

Considering the possible mechanisms underlying mycotoxin production inhibition by lipopeptides, it is worth noting the work of Hu et al.^41^. They demonstrated that inhibition of fumonisin B1 (FB1) synthesis can occur through transcriptional regulation of FUM1 and FUM8, the latter encoding a critical enzyme in the fumonisin biosynthetic pathway. Fengycin treatment led to complete suppression of FUM1 expression and significant down-regulation of FUM8. By analogy, it is plausible that lipopeptides from the genus *Pseudomonas* , including amphisin, may act similarly by regulating mycotoxin synthesis through down-regulation of key biosynthetic genes.

Concluding our research as well as the mentioned studies of other authors expanding the spectrum of mycotoxins, which production can be controlled using bacterial biosurfactants, thus highlighting the potential use of lipopeptides and rhamnolipids for crops biocontrol.

## Conclusions

In this paper, the antifungal activity of biosurfactants synthesized by *P. fluorescens* DSS73 and *P. aeruginosa* #112 was assessed. The performed studies demonstrated the significant potential of biosurfactants to inhibit the growth and development of pathogenic fungi belonging to the genera *Aspergillus*, *Botrytis*, *Colletotrichum*, *Fusarium*, *Monilinia*, and *Penicillium*. In addition, biosurfactants were found to reduce the production of sterigmatocystin by *A. amoenus* DSM 1943 and *A. quadrilineatus* DSM 820, highlighting their potential use in post-harvest crop protection and sustainable agriculture. The results suggest that biosurfactants produced by bacteria of the genus *Pseudomonas* may serve as effective and environmentally friendly agents for the control of pathogenic fungi.

Future research should include the analysis of the effects of biosurfactants on plant tissues under *in vivo* conditions and their application in various biological and industrial systems. The scalability of biosurfactant production is a crucial factor for practical implementation. The use of food industry by-products such as black cumin cake, molasses, and corn steep liquor as substrates offers a cost-effective and sustainable route for large-scale synthesis, reducing overall production expenses and environmental impact. Moreover, integration of biosurfactants into existing agricultural systems may occur through their formulation as foliar sprays, seed coatings, or soil amendments, enabling their combined use with conventional biocontrol and fertilization strategies. However, field deployment requires further evaluation of compound stability under varying temperature, UV exposure, and microbial activity, as these parameters can influence antifungal efficiency over time. Therefore, future studies should focus on formulation optimization and controlled field trials to ensure long-term stability and efficacy of biosurfactants under natural agricultural conditions.

Overall, potential applications of these compounds in biotechnology, agriculture, and medicine open new, environmentally friendly avenues for the sustainable control of pathogenic fungi.

## Materials and methods

### Microorganisms and culture conditions

An amphisin-producing strain *P. fluorescens* DSS73^19^ was graciously provided by Dr. Ole Nybroe (University of Copenhagen, Denmark), while rhamnolipid producing *P. aeruginosa* #112^42^, was isolated from an oil sample taken from an oil field in Brazil. Bacteria were stored at the Department of Biotechnology and Food Microbiology, Wrocław University of Environmental and Life Sciences (Wrocław, Poland) as a glycerol stock (20% v/v) at − 80 °C. In order to prepare inoculum bacteria was cultured at 37 °C, overnight (180 rpm) in Luria-Bertani (LB) medium (A&A Biotechnology, Gdańsk, Poland) composed of (g/L): NaCl 10.0; tryptone 10.0; yeast extract 5.0; pH = 7.0.

The fungal strains *A. quadrilineatus* DSM 820, *A. amoenus* DSM 1943, *C. coccodes* DSM 62126, *F. culmorum* DSM 1094, *P. nordicum* DSM 12639 were obtained from the German Collection of Microorganisms and Cell Cultures GmbH (DSMZ, Braunschweig, Germany). *B. cinerea* IOR 663 and *M. fructigena* IOR 2138 were obtained from the culture collection of Institute of Plant Protection National Research Institute (Poznań, Poland). *P. expansum* CBS 230.38 was purchased from the Centraalbureau voor Schimmel cultures (CBS, Utrecht, The Netherlands). Fungi were cultured on Potato Dextrose Agar (PDA) medium (A&A Biotechnology, Gdańsk, Poland) composed of (g/L): potato extract 4.0, dextrose 20.0, agar 15.0; pH = 5.6 at 25 °C for 7 days and stored at 4 °C for a maximum of 3 weeks. When needed, strains were passaged onto fresh PDA medium plates.

### Production, purification and characterization of amphisin

The production and purification of amphisin were performed as described previously by Ciurko et al.^22^. In brief, the biosurfactant was produced under optimal conditions in 300 mL Erlenmeyer flasks containing 50 mL of medium composed of 6.6% black cumin cake (AleOlej.pl, Wrocław, Poland) and 8.0 mM NaCl solution (POCH, Gliwice, Poland). Medium was inoculated with 0.5 mL of *P. fluorescens* DSS73 LB-culture. Biosurfactant production was performed at 28 °C for 6.5 days at 180 rpm. Finally, the post-culture medium was centrifuged at 9500 × g for 20 min to separate biomass and supernatant.

Amphisin isolation was conducted using adsorption chromatography techniques. Column filled with Amberlite XAD-2 sorbent (Sigma-Aldrich, St. Louis, MO, USA), was conditioned according to the producer instructions. Culture supernatant was applied onto the column at a constant flow rate of 5 mL/min. Then, the system was washed with 500 mL of phosphate–buffered saline buffer (PBS, pH 7.0; Merck, Darmstadt, Germany) to remove unbound particles. Finally, bounded amphisin was eluted with methanol in a volume of approximately 500 mL at the same flow rate.

Amphisin methanol solution was evaporated using a vacuum evaporator at 150 mBar and 40 °C to dry. Then, the crude biosurfactant was dissolved in a small amount of distilled water and applied to Chromabond C18 SPE system for additional purification (Macherey-Nagel, Düren, Germany). Amphisin was eluted using a gradient of acetonitrile-water (20%, 50%, 80%, 100% (v/v)) and the solvent was successively evaporated using a vacuum evaporator under the same conditions as described above. The purification process was monitored through high-performance liquid chromatography (HPLC) using Ultimate 3000 HPLC system from Thermo Fisher Scientific (Waltham, MA, USA) equipped with a Hypersil GOLD column (5 μm; 4.6 × 150 mm). As the mobile phase, solvent A (0.1% trifluoroacetic acid) and B (0.1% trifluoroacetic acid in acetonitrile) were applied according to the following scheme (% A:B v/v): 0 min (50:50), 5 min (20:80), 9 min (10:90), 15 min (0:100), 21 min (0:100), 24 min (50:50) and 25 min (50:50). Tested samples, injected onto a column in the amount of 10 µL, were eluted for 25 min with a flow rate of 0.5 mL/min and detected at the wavelength of 210 nm. The purified amphisin was lyophilized and stored at − 20 °C for further studies. Mass spectrometry (*data not shown*) of the purified amphisin revealed over 99% purity.

### Production, purification and characterization of rhamnolipids

The production of rhamnolipids by *P. aeruginosa* #112 was carried out in 500 mL Erlenmeyer flasks containing 200 mL of medium composed of 10% corn steep liquor (Sigma Aldrich, St. Louis, MO, USA) and 10% molasses (Sojaprotein, Bečej, Serbia). Medium was inoculated with 2 mL of LB bacterial culture. Rhamnolipids synthesis were carried out at 28 °C for 120 h at 180 rpm. Successively, the culture media were centrifuged (10000 × g, 20 min) to separate culture supernatant, which was purified and analyzed in the same manner as described in section entitled “*Production*,* purification and characterization of amphisin*”. The relative abundance of each homolog was determined by calculating the area under the chromatogram signals.

In addition, the individual rhamnolipid in the provided mixture were identified using liquid chromatography coupled with ion trap/time-of-flight mass spectrometry (LCMS-IT-TOF) technique. A sample of rhamnolipids was dissolved in a water-acetonitrile mixture (50:50). The LC-MS analysis was performed on a Shimadzu IT-TOF instrument with an electrospray ion source in negative ion mode. Separation was carried out on an Aeris PEPTIDE 3.6 μm XB-C18 column (50 × 2.1 mm) with a gradient elution from 5 to 90% B in A (A = 0.1% HCOOH in water; B = 0.1% HCOOH in acetonitrile) at room temperature for 15 min (flow rate: 0.2 ml/min). Mass spectrometry of the purified rhamnolipids revealed over 98% purity.

### Antifungal activity assay

The antifungal properties of biosurfactants produced by *P. fluorescens* DSS73 and *P. aeruginosa* #112 were tested in relation to *A. quadrilineatus* DSM 820, *A. amoenus* DSM 1943, *P. expansum* CBS 230.38, *P. nordicum* DSM 12639, *B. cinerea* IOR 663, *C. coccodes* DSM 62126, *F. culmorum* DSM 1094, *M. fructigena* IOR 2138. Assay was performed in 55 mm diameter petri dishes containing 8 mL PDA medium, supplemented with lyophilized biosurfactants. Amphisin or rhamnolipids were added to PDA media, as a powder, at the concentration of 0.3 and 0.5 or 1.5 and 3.0 g/L, respectively and subjected for autoclave sterilization. PDA media were cultured with a disc of mycelium (8.8 mm in diameter), obtained using cork borer from PDA inoculation cultures, applied centrally. PDA media without the addition of biosurfactants were served as a control. Fungal cultures were incubated at 25 °C until the entire surface of the control plates was covered by mycelium.

Biosurfactants activity were evaluated after 48 h and 72 h of incubation and then with the 48 h intervals by comparing the diameter of the fungal growth on the control and tested medium. Mycelium inhibition rates was calculated according to the formula:

$$\:Growth\:inhibition\:x\left(\%\right)=\left(1-\frac{diameter\:x}{diameter\:c}\right)\times\:100$$

where *diameter x* (mm) is the mycelial growth on the medium containing the biosurfactants, and *diameter c* is the diameter of mycelial growth on the control plate.

### Sterigmatocystin production analysis

Sterigmatocystin is a polyketide mycotoxin, which production so far has been determined for several fungal species belonging to the genera *Aspergillus*, *Bipolaris*, *Botryotrichum*, *Humicola* and *Penicillium*. Sterigmatocystin has been isolated by the first time in 1954 from the culture of *A. versicolor.* However, it was just as frequently isolated from another *Aspergillus* cultures such as *A. flavus*, *A. parasiticus*, *A. nidulans*. The sterigmatocystin shares its biosynthetic pathway with the aflatoxins, which are considered the most potent carcinogenic mycotoxins known. Particularly, sterigmatocystin acts as biogenic precursor of aflatoxin B1 and aflatoxin G1^43^.

In this study, the effect of amphisin and rhamnolipids on the production of sterigmatocystin were analyzed according to *A. amoenus* DSM 1943 and *A. quadrilineatus* DSM 820. Fungal culture, obtained as described in a section entitled “*Antifungal activity assay”*, were cut into pieces, transferred to a 50 mL eppendorf tube and extracted with 20 mL of a mixture of acetonitrile, methanol and acetic acid applied in relation 78:20:2, v/v/v. Mycotoxin extraction was performed by agitation of the media at high speed for 10 min, followed by the incubation at room temperature in the dark for an hour.

Subsequently extracts were subject in a value of 10 µL into HPLC analyses performed with Ultimate 3000HPLC system (Thermo Fisher Scientific; Waltham, MA, USA) equipped with a Hypersil GOLD (5 μm, 4.6 × 150 mm) column. Elution was performed at 40 °C with an isocratic solvent system being a mixture of acetonitrile and water (60:40v/v) with a flow rate 1.0 mL/min. Sterigmatocystin was detected at a wavelength of 246 nm. Each sample was subject to the three-step extraction to verify, whether the entire content of mycotoxin was remove at the first stage. In order to prepare the calibration curve, a series of sterigmatocystin dilutions in the concentration range between 10 and 0.1 µg/mL was prepared using the standard (Sigma Aldrich, St. Louis, MO, USA). Calibration curve constructed for sterigmatocystin was characterized by high linearity (R^2^ = 0.9896).

## Data Availability

The datasets generated during and/or analysed during the current study are available from the corresponding author on reasonable request. tomasz.janek@upwr.edu.pl.
